# Illustrations of the Reciprocal Sympathies between the Uterus and Bladder in Woman

**Published:** 1858-07

**Authors:** W. F. Montgomery


*Illustrations of the reciprocal sympathies between the uterus and bladder in woman.* By W. F. Montgomery, M. D., read before the Medical Association of the College of Physicians, 4th November, 1857.

It is familiar to us all, that in the human female there is an intimate reciprocal sympathy between the uterus and bladder, and other parts of the urinary apparatus ; so that, under a variety of circumstances, when the former organ is the seat of any anomalous action, or brought into a state of exalted sensibilty, whether from natural or morbid causes, the latter is not only liable, but very apt to sympathize, and suffer correspondingly.

This is constantly exemplified in the increased urinary irritation so often accompanying ordinary healthy menstruation, and still more remarkably, when the latter function happens to be painfully performed. Again, in early pregnancy, the same thing is observed ; and the remark is trite, that morbid actions in the uterus, whether benign or otherwise, often have the earliest announcement of their invasion, in symptoms of disturbance first noticed in the functions of the bladder.

Thus, congestion or slight ulceration of the cervix uteri, and still more strikingly, malignant affections of that part, frequently excite, in the first instance, in the patient's mind, only apprehensions of gravel, or some vesical disease, for which alone she is induced to seek advice; but woe betide us, in this and many other circumstances, if we let ourselves be beguiled into the belief that because a particular organ or locality is affected with certain anomalous symptoms, it is therefore the seat of some disease, of which these symptoms are to be taken as indications; and so prescribe. Could we, for instance, expect to cure the itching of the nose and angle of the eye which accompanies the presence of intestinal worms, by applications to the Schneiderian, or conjunctival membrane?

Mrs. C. had, for a long time, intense, intolerable, distracting itching of the perineum and anus, which really rendered her life miserable, and for which she had consulted many, and used a multiplicity of remedies; many of them, no doubt, very appropriate for pruritus, but not for her. When she came under my care, I also, at first, adopted the wrong course; I prescribed for the symptom, and not for its cause. But fortunately, after seeing her a few times, something led me to suspect the existence of intestinal worms. I gave her a dose of the kousso, which caused the expulsion of some very large lumbrici, and all her troubles were forthwith at an end.

Mrs. M. consulted me for pruritus of the pudendum, from which she suffered to such a degree, and it was accompanied with other symptoms of so distressing a kind, that she declared she loathed herself, and felt her life an intolerable burthen to her. She had used gallons of lotions, and all sorts of ointments, without the slightest relief. Examination showed an intense congestion of the cervix uteri; this was made the object of treatment, and on its removal, the pruritus and its miserable concomitants totally ceased.

I have already called the consent between the uterus and bladder a reciprocal sympathy, because it equally acts in the reverse direction, irritations of the bladder being frequently found to influence and disturb the functions of the uterus—a fact which should not be forgotten in practice, and especially in the treatment of the diseases of pregnancy, when the administration of the more irritating diuretics should be avoided, lest they should excite contraction of the fibres of the uterus, and so induce premature expulsion of its contents. That they are capable of so doing is, unfortunately, popularly known, and advantage is taken of such knowledge for base and wicked purposes.

And again, we must remember that vesical disturbances may produce a group of symptoms so closely resembling those arising from disease of the uterus, as to be mistaken for them.

Several years ago, there occurred in this city a case forcibly illustrative of this, and which excited no small sensation. The wife of a general officer, at that time holding the highest military command in this country, began to complain of distressing symptoms, having all the characters of those produced by uterine disease. Such was her own conviction, and on her consulting an accoucheur, then in large and high practice, her worst fears were confirmed; he pronounced the affection to be cancer uteri, and could only promise palliation- But she had many anxious friends, whose happy privilege it is always to hope for the best, and some of them urged upon her the necessity of having another opinion; to this she at last consented, and the gentleman called in pronounced the case to be one of stone in the bladder; the stone was extracted, and the lady passed at once from a state of pain and misery to one of comfort and happiness.

A few years since, a patient came to consult me, stating that, to gratify her friends, she had come to town for my advice, although quite aware that she could not be cured. She also handed me a written statement of her case, which set forth that she had had seven labors of terrible severity, owing to contracted pelvis, always requiring instrumental delivery; that for some months she had exhibited unequivocal symptoms of the existence of cancer uteri; and I confess that, from this account and the woman's own description of her symptoms, I thought there was little room for doubt as to the nature of her malady. However, I, of course, gave no opinion, and suspended my judgment until I should have instituted a careful examination; on doing so, I could discover no disease of the uterus, but the neck of the bladder was distended and felt very hard. I passed a sound into it, which at once struck against a stone of considerable size. Mr. Fleming now saw the case with me, the stone was removed, and the woman soon returned home well, and continued so.

It is not my intention to enter into a lengthened exposition of all the details of this part of our subject, but only to ask the attention of this distinguished meeting for a few minutes, while I review, as briefly as may be, some forms of disturbance of the functions of the bladder occurring at the time of labor. And I may just premise, that considering the frequency with which we witness such interchange of sympathies as we have been hitherto discussing, and their production, in many instances, from comparatively unimportant causes, we would anticipate from the close relation of juxtaposition between the uterus and bladder, and from the direct exposure of the latter organ to the powerful mechanical efforts cf the uterus, as well as from the marvelous anatomical and physiological changes taking place in the latter, during pregnancy and parturition, and immediately after—we would, I say, be led to expect that the innervation and powers of the bladder would be likely to exhibit still more marked and decided evidences of impairment, or some other abnormal condition, in the puerperal woman; and accordingly, in practice, we find that there are, at least, four forms of disturbance of the functions of the bladder, which are not unfrequently produced by parturition, even when that process is easy and natural:

1st. Irritability, causing an inconveniently frequent desire to discharge its contents; and this with a certain amount of pain.

2d. Loss of expulsive power; the natural sensibility being unaffected.

3d. Total loss of the natural sensibility, or irritability, which prompts to the evacuation of the contents of the organ.

4th. A peculiar form of hysterical retention.

The first two of these states require little more than to be glanced at; the third and fourth demand a somewhat more particular consideration.

1st. The irritability of the bladder which succeeds labor, almost always, yields readily to soothing measures, such as warm anodyne fomentations, a linseeed meal poultice, opiate and camphorated embrocations, an opiate suppository in the rectum, or the administration of the mist. camphoræ c. magnesia, with tincture of hyoscyamus and syrup of poppy. Should there be reason for suspecting inflammation, it may be necessary to apply a few leeches, and use other appropriate remedies; but this is rarely the case.

2d. When the expulsive power is in abeyance, but the natural sensitive irritability remains unimpaired, the desire to evacuate the contents of the organ becomes distressingly urgent, in proportion to the amount of accumulation within it.

This condition is sometimes produced by pressure from the uterus happening to be larger or lower than usual, perhaps displaced, or from the presence of an uterine tumor, in which case, raising up the obstacle may remove all difficulty. Sometimes, merely permitting the patient to assume the sitting position may suffice. Should these plans not succeed, the introduction of the catheter gives the desired relief.

3d. Again, there is the third and most important variety, in which there is no irritability, not even the natural desire to evacuate the urine, nor sensation, nor consciousness of requiring to do so, although a large accumulation is taking place, which will soon produce symptoms, not alone very distressing in themselves, but, what is worse, conditions which may lead us greatly astray, and excite groundless apprehensions of great impending danger; and it will not lighten our discomfort, should it turn out, as has often happened, that the patient owes all her annoyance to our inadvertence, or the carelessness of the nurse-tender; for if we are careful in making the requisite enquiries, and give the proper directions to the nurse, and she attend to them, the accident, of which I am about to speak could not happen.

The first time I remember to have had my attention drawn to this condition was in the year 1832, and in the case of an esteemed medical friend's wife; who was delivered of her first child, after a natural labor of fifteen hours, presenting nothing unusual, if we except the most tremendous rigor I ever witnessed, which occurred shortly after delivery, and was so violent that I really thought it must have ended in convulsions; but simple means removed it, and she went on well. Before leaving her. I cautioned the nurse to encourage the lady to pass water in the course of the evening, as she had not done so for several hours; this, however, was neglected, as the lady neither expressed, nor felt any want; and, at my next visit, I found my patient very feverish, with headache; a pulse above 120; the belly tumid and tender to the touch. Before proceeding to introduce the catheter, of which she had a great horror, I advised her trying to pass water, which she did, to the amount of at least two quarts, with instant relief to all the symptoms. Her convalescence was uninterrupted.

At a time when there were occurring several cases of puerperal fever through town, a medical friend called on me to request that I would accompany him to visit a patient of his, who had been confined two days previously, after a severe and protracted labor. She was not going on well, and he thought he had to deal with a case of the above mentioned formidable disease, as his patient was very feverish, had most distressing headache a rapid pulse, with abdominal pain, swelling and tenderness.—On seeing her, I thought the presence of a large quantity of fluid was beyond doubt; a catheter was introduced, and an enormous accumulation of urine withdrawn, with immediate and decided relief to all the symptoms. It was then ascertained, that she had not passed water since her confinement, nor for many hours before it; she recovered perfectly.

The next case occurred to me under circumstances which invested it with a very unusual interest, and caused it to make a great impression on me. I happened to be staying for a few days in a very fashionable watering place in England, where there resided a family of great wealth and consideration in society; the eldest son of which had married a lady of fortune and high connection. This lady had recently been confined of her first child; and, as may be readily supposed, the attendance upon her had been a matter of warm ambition among the local practitioners; and the gentleman who carried off the prize was, I fancy, more envied than congratulated by his confreres.

I met this gentleman accidentally in society, and the following morning he called on me in a state of painful agitation and distress; "I am," he said, "in a terrible dilemma, and fear I am a ruined man." He then proceeded to tell me that he had attended Mrs. *** three days before; that she had rather a severe labor; and that at the end of thirty hours, finding the labor not likely to terminate by the natural efforts, the head having remained stationary for several hours low down in the pelvis, and pressed strongly against its floor, he had delivered her with the forceps, without, as he assured me, any difficulty whatever. All then seemed well; but next day, the lady was uncomfortable, restless, feverish, and rather larger than she ought to be, and this state went on increasing until the third or fourth day, when, to his horror, the urine began to trickle away incessantly, and a slough, about the size of a sixpence, was discharged from the vagina. He at once, naturally enough, concluded that a vesical fistula was established; and I, taking his account as my guide, thought there was but little doubt that his worst apprehensions would be realized.

At his urgent request, I accompanied him to visit the lady, whom I found with a hot skin, much headache, a very quick pulse, a very distended abdomen, which was moreover so tender that she could hardly bear it to be touched; but I distinctly ascertained the presence of fluid. On examining per vaginam, I could not detect by the finger any breach of surface along the anterior wall, or back of the bladder, and I suggested to Mr. *** that it would be well to pass a catheter into the bladder; which, at his request, I did; and gave exit to a quantity of urine, sufficient nearly to fill an ordinary wash-pan basin. Subsidence of the abdominal swelling immediately took place, the lady felt inexpressible relief, and from this day went on well.

The truth was, that the bladder had been forgotten by all the parties concerned; and the patient had never passed water since her confinement, nor felt a desire to do so, until at last the bladder became so distended, that the resistance at its neck was overcome, and the urine leaked out at the front as fast as it was pumped in from the ureters at the back; and it so happened that, just as this began, a small slough had separated from the mucous membrane of the posterior wall of the vagina, which had been strongly pressed on by the head for several hours.

4th. Another state, not exactly ranging under any of the former kinds, seems to be of a purely nervous or hysterical character, and mixed up with a certain amount of mauvaise honte. The patient has a decided desire to pass water, and suffers distress from its retention; but has, at the same time, the greatest reluctance to make the neccessary effort, and positively refuses to try, if any one, even the nurse-tender, is present, declaring that it would be impossible for her to succeed.

Under these circumstances, suitable arrangements should be made by the nurse, and the patient then left by herself, for a time, during which she may succeed in accomplishing the desired object; if she does not, some anti-nervous medicine should be given, with strong assurances of its potency in removing such difficulties; let her try again during our absence, and if she has not succeeded when we come to pay our next visit, we must declare that longer delay would be unsafe, that we will wait a little while in the drawing-room, and if she does not then succeed, that we must draw off the water before we leave the house; this generally insures success.

In many of these cases they do not succeed, because they do not make the proper effort; and this, I believe, is oftentimes, simply because they cannot, and not always because they do not, choose; this state of nervous inability ought to meet with much tender consideration. I may observe here, that I have never met with this peculiar form of affection, except in the higher grades of society, and almost always in women of a highly sensitive nervous temperament, some of them have experienced a similar difficulty before marriage, and also under ordinary circumstances distinct from pregnancy.

Now, is this state which I have just been attempting to describe, analogous to, or identical with another, which I have a few times met with in practice, and which may be thus described?—A lady in perfect health retires to her bed room for the night, and before lying down to rest, attempts to make water, and finds she cannot—she is much surprised—goes to bed, and perhaps falls asleep; in the morning, she is in great distress; but still unable to empty the bladder, and now her pain is so great, she is compelled to seek for assistance—the catheter is introduced, and all her trouble is at an end; or, perhaps, for several days, its use continues to be required, and then all goes on as well as ever; but, in either case, no circumstance of general ill health, or local derangement or displacement, can be discovered. The woman, in fact, is otherwise quite well.

The year before last a married lady of a highly nervous temperament, so affected, drove six miles into town to a house, in great torture: I drew off the water, and she required no further assistance.

Last year, I was urgently summoned to see an equally nervous maiden lady similarly situated; on laying my hand on the abdomen, I felt a tumor, as large as a melon, and as hard as a cricket ball; and per vaginam, it really, at first, suggested the idea of a fibrous tumor of the uterus, this organ being quite displaced. I introduced the catheter, drew off a large accumulation of urine, and the abdominal tumor—which was nothing more than the distended bladder—at once disappeared. The feature in this case which particularly arrested my attention was, the extraordinary hardness and the distinct outline of the abdominal tumor, which would readily have caused it to be mistaken for a solid morbid growth.

I may here observe, that in the well known case of the virgin mock prophetess, Joanna Southcott, there was felt, by competent judges, a circumscribed tumor in the abdomen, which was supposed to be the gravid uterus. Dr. Reese, in his published history of the case, says: "In that part occupied by the womb, there was a firm circumscribed tumor as large as a man's head, bearing the shape of the womb; I had no doubt of its being an enlargement of that organ." But when she died, no tumor existed, and that which was felt during life was attributed to the prophetess having learned to retain the urine until the bladder became considerably distended; which seems highly probable.

In my case last related, it was necessary to relieve the lady every day for a weak, when she perfectly regained the power of micturition. I may just mention, that the remedy which seemed to remove her inability was the ergot of rye. Did it do so by stimulating the fibres of the uterus in the first instance, and then, by consent, those of the bladder? Perhaps so; but as the same agent has produced a similar effect in men, a direct influence may be equally admitted.—*Dublin Hospital Gazette—Virginia Medical Journal.*

